# Mapping hazards to the global food system

**DOI:** 10.1007/s10661-024-13475-4

**Published:** 2024-12-04

**Authors:** David F. Willer, Samuel Short, Diana Khripko, Silviu O. Petrovan, Alec P. Christie, Julie Bremner, William J. Sutherland, David C. Aldridge

**Affiliations:** 1 Department of Zoology, University of Cambridge, The David Attenborough Building, Pembroke Street, Cambridge, CB2 3QZ UK; 2 Institute for Manufacturing, IfM Engage, University of Cambridge, 17 Charles Babbage Road, Cambridge, CB3 0FS UK; 3 Maastricht Sustainability Institute,, Maastricht University, P.O. Box 616, 6200 MD Maastricht, Netherlands; 4 Centre for Environment, Fisheries and Aquaculture Science, Pakefield Road, Lowestoft, NR33 0HT UK; 5 Collaborative Centre for Sustainable Use of the Seas, School of Environmental Sciences, University of East Anglia, Norwich Research Park, Norwich, NR4 7TJ UK

**Keywords:** Risk management, Monitoring, Security, Agriculture, Aquaculture, Fishing

## Abstract

Environmental hazards associated with the global food system threaten societal integrity. Yet, there is a major data gap in the global understanding of how the prevalence of hazards is changing over time, how different classes of hazard are distributed, and whether the combined literature represents hazard prevalence equitably across research, policy and legislation, and news. Here, we explore this data gap, leveraging global research, policy, and news databases. We reveal increasing attention on food system hazards over time, in line with major geopolitical events. Coverage on environmental hazards is not distributed equally geographically, and media attention does not match research and policy evidence focus. Climate change and water scarcity in particular receive substantial attention across all source types, whilst, for example biodiversity loss, genetic erosion, or harmful algal blooms receive much less. Environmental, financial and food systems sustainability damage due to hazard neglect should be avoided and a first step is to understand, map, and quantify biases in focus.

**Supplementary Information:**

The online version contains supplementary material available at 10.1007/s10661-024-13475-4.

## Introduction

In an era marked by unprecedented interconnectedness and rapid environmental change, the resilience of the global food system stands as a paramount concern (Fanzo et al., 2021; Johan et al., 2020; Savary et al., 2020). In order to improve the resilience of our food system, there is an urgent need for better data and understanding of the multifaceted environmental hazards across the intricate web of production, distribution, and consumption in food systems (Tendall et al., 2015). We define a food-system hazard as any biological, chemical, physical, or socio-economic factor acting on food production and distribution that prevents resources being turned into safely consumable food, or factors associated with food production that degrade the natural environment or contribute to climate change. Food systems are thus both a source and a sink for hazards, generating environmental challenges such as greenhouse gas emissions and, in turn, impacted by hazards such as pests and pathogens (Bremner et al., 2023). This complexity renders hazard management difficult and threatens the integrity of food supply.

Globally, numerous parallel efforts are being made to collect and collate data on hazards and improve understanding of our food system and the complex interactions within it. Prominent examples referring to specific components of the global food value chain include the WWF Water Risk (WWF, 2024b) and Biodiversity Risk Filters (WWF, 2024a), The Global Food Security Index (Economist, 2024), and Food Systems Dashboard (GAIN, 2024). Our World in Data has collated an extensive range of global summaries on various aspects of the global food system, ranging from hunger and undernourishment to crop yields to animal welfare (GlobalChangeDataLab, 2024). In addition, new frameworks are being proposed to assess risk, and these highlight previously overlooked components including risks that both arise from climate change and our responses to climate change (Simpson et al., 2021).

Despite the growing attention to risks in the food system, critical under-researched questions on environmental hazards remain. Research, policy, and the media all identify that we have been undergoing, and continue to undergo, a phase of rapid global environmental change and the emergence of new or shifting hazards (Chakraborty & Maity, 2020). Yet, we lack a systematic and quantitative understanding of how hazards to the global food system are changing over time, and what key policies or changes might be associated with this. There is also an urgent need for an improved understanding of how hazards are distributed globally and how hazard prevalence varies by region; these are critical aspects to design more effective intervention and mitigation strategies and proactive policy responses. There are innumerable case studies on specific regions that will be worse affected by specific hazards, such as Mediterranean agriculture with rapidly increasing water scarcity (Rocha et al., 2020), finfish aquaculture in South America with changes in the prevalence and severity of the El Nino Southern Oscillation (Gasalla et al., 2017; Yáñez et al., 2017), or zoonotic diseases originating from birds and other wildlife in Southeast Asia (Wikramanayake et al., 2021), alongside proposed tools to track these risks. However, a comprehensive inventory allowing fair comparisons between global regions is not available. Crucially, there is a need to overcome a major issue regarding the scientifically quantified versus publicly perceived presence of hazards. Ideally, academic research would reflect the regional and global prevalence of global hazards, and this would be reflected in policy and legislation, with the media then accurately portraying this, but the reality is that this does not happen; there are myriad biases in the translation of information in order to meet specific political, industrial, or commercial agendas (West & Bergstrom, 2021; Willer et al., 2019). The concern here is that certain hazards are prioritised at the expense of other perhaps equally, or even more, significant threats in the food system, with potential far-reaching consequences for long-term food availability, food security, and environmental resilience.

In this study, we aim to answer these key questions of environmental hazards to and of the food system regarding changes over time, regional distribution, and perception versus reality. This is now possible via the availability of global databases yet to be explored formally for food-system hazards. These include Scopus, the world’s largest comprehensive academic research database (Elsevier, 2024), the Food and Agriculture Organisation of the United Nations policy and legal database (FAO, 2024), and the ProQuest media database (Clarivate, 2024). These tools allow us to achieve not only global breadth but also specific depth in our analysis, an important aspect given that each global region has its own unique vulnerability profile influenced by a multitude of factors including physical landscape, climatic conditions, and sociopolitical dynamics (Birkmann et al., 2022). Our analysis highlights key matches in hazard prevalence and attention, with research, policy, and media hazard reports all increasing over time in correlation with major global events, but also key mismatches, with a select few hazards receiving the majority of attention, and others very little, particularly from the media.

## Methods

### Environmental hazard and search term inventory development

A systematic process was used to build a comprehensive list of environmental hazards from the food system, and environmental hazards on the food system to provide a backbone for the global hazard mapping work. This consisted firstly of developing a top-level value-chain perspective on the major segments in the global food system—covering agricultural input supply, plant agriculture, terrestrial animal farming, hunting, aquaculture, wild fisheries, food processing, packaging, trade, distribution, consumption, and waste disposal. This structure then informed the selection of specific search terms to facilitate a rapid but comprehensive search of the peer-reviewed and grey literature to identify relevant environmental hazards within each segment. This study excluded human nutrition and health, food waste, and socio-economic hazards. Information was extracted for 337 environmental hazards spread across 16 segments of the food system. This list was then consolidated in collaboration with experts from the Centre for Environment, Fisheries and Aquaculture Science (Cefas) and Animal and Plant Health Agency (APHA) to agree upon common wording, resulting in a working list of 39 direct hazards and a further 11 indirect hazards. These hazards were then sub-categorised under the commonplace terminology of biological, chemical, and physical hazards in line with Hazard Analysis Critical Control Point (HACCP) Guidelines (FDA, 2024). To facilitate more efficient academic, policy, and media database interrogation, this list of environmental hazards to the food system was consolidated into a shorter list of 26 hazards using an expert solicitation process within the University of Cambridge and Institute for Manufacturing expert pool. Using the same expert approach, a selection of 6 sub-search terms was generated for each hazard, aside from four hazards where 5 terms were deemed most appropriate. The list of 26 hazards is as follows, and the sub-search terms are available in Supplementary Table 1: antibiotic resistance, biodiversity loss, genetic erosion, harmful algal blooms, human–environment conflict, invasive non-native species, pathogens, pests, weeds and poisonous plants, zoonotic diseases, air pollution, chemical pollution, fertilisers, herbicides, pesticides, plastics in food production, radiological contamination, water pollution, climate change, destructive fishing practices, flooding or water logging, land and water use change, noise and light pollution, soil degradation, water scarcity, wildfires.

### Data extraction

Data on academic research articles over the past 20 years (2004–2023 inclusive) were extracted from Scopus (Elsevier, 2024). Each of the 26 hazards was searched using their corresponding sub-terms, in the title, keywords, or abstract of the articles. Additional search term filters were applied to ensure results were specific to the food system rather than all fields of academia, using operators for ‘hazard’ or ‘risk’ alongside ‘food system’ or ‘agriculture’ or ‘food production’ or ‘farming’, ‘aquaculture’ or ‘fisheries’ or ‘livestock’. The number of research articles corresponding to each hazard for each year and each global region was counted. To account for increasing general academic publication rates over time, data were baselined with the annual number of publication records found using the broad search term ‘food’. In total over 20,000 academic records were extracted from Scopus relating to hazards and risks in the food system.

Policy and legislative data were extracted for the years 2004–2023 inclusive from the FAOLEX database (FAO, 2024). The same 26 hazard search terms and their sub-search terms were used as for the Scopus search, albeit without a food system filter, given the FAOLEX database is already specific to food. To account for increasing policy publication rates over time, data were baselined against the total annual publication records in the FAOLEX database of about 240,000 records. The number of policy and legislative documents for each country and year related to the hazards was counted, with a total of 19,000 records extracted.

News data for 2004–2023 were extracted from ProQuest (Clarivate, 2024). The same search format was used as for the Scopus search, including the same filters to ensure specificity to the food system, and the search was made specific to news articles and editorials. These data were deemed inappropriate to baseline or for the inclusion of an additional “hazard” or “risk” search operator. In summary over 11,000 records were extracted.

Data were extracted using the English language versions of the sub-search terms. This was justified on the grounds that ProQuest searches multiple languages automatically, including English, Spanish, Portuguese, German, Italian, Russian, Chinese, and Japanese (Clarivate, 2024), that FAO policy and legislation documents have English versions by default (FAO, 2024), and that the vast majority of research articles have an English title, abstract, and keywords (Elsevier, 2024). We note that the selection of 6 search terms across research, policy, and media represents a compromise. Given that media is by definition less technical in reporting, a more diverse range of terminologies may be used and so articles may not be picked up—one hypothetical example might be ‘walk outs’ to describe lobster die-offs related to algal blooms, rather than referring to algal blooms directly (Stephen & Hockey, 2007). Yet broadening the range of terms leads to more false positives in the search, hence the selection of six sub-search terms per hazard. It is appreciated that inevitably some false positives will have been picked up on large dataset such as used in this study. We also highlight that in many cases, a given research, policy, or media document would refer to two or more hazards. In these cases, the document would be counted in each hazard category, given that there was no irrevocable way of deeming a specific document was primarily focussed on one specific category. The six sub-search terms were carefully selected to minimise the chance of documents being double-assigned or inappropriately to another hazard. For example ‘Species Range Shifts’, which might have been an appropriate term to search for ‘Invasive Non-Native Species’, was not used as a search term for ‘Invasive Non-Native Species’ as it might pull up too many results that were focussed on climate change and not invasive species. We acknowledge that the ‘Climate Change’ hazard does include ‘Climate Change’ as a sub-search term (alongside ‘Extreme temperature’, ‘Extreme weather’, ‘Sea level rise’, ‘Ocean acidification’, and ‘Global warming’, Supplementary Table 1), even though ‘Climate Change’ may sometimes be used in documents to refer to other hazards related to anthropogenic activity. It was however deemed that the exclusion of ‘Climate Change’ as a search term would lead to a greater negative impact from relevant documents being missed than the potential impact of hazard misassignment.

## Results

### Hazard publications over time

**Fig. 1:**
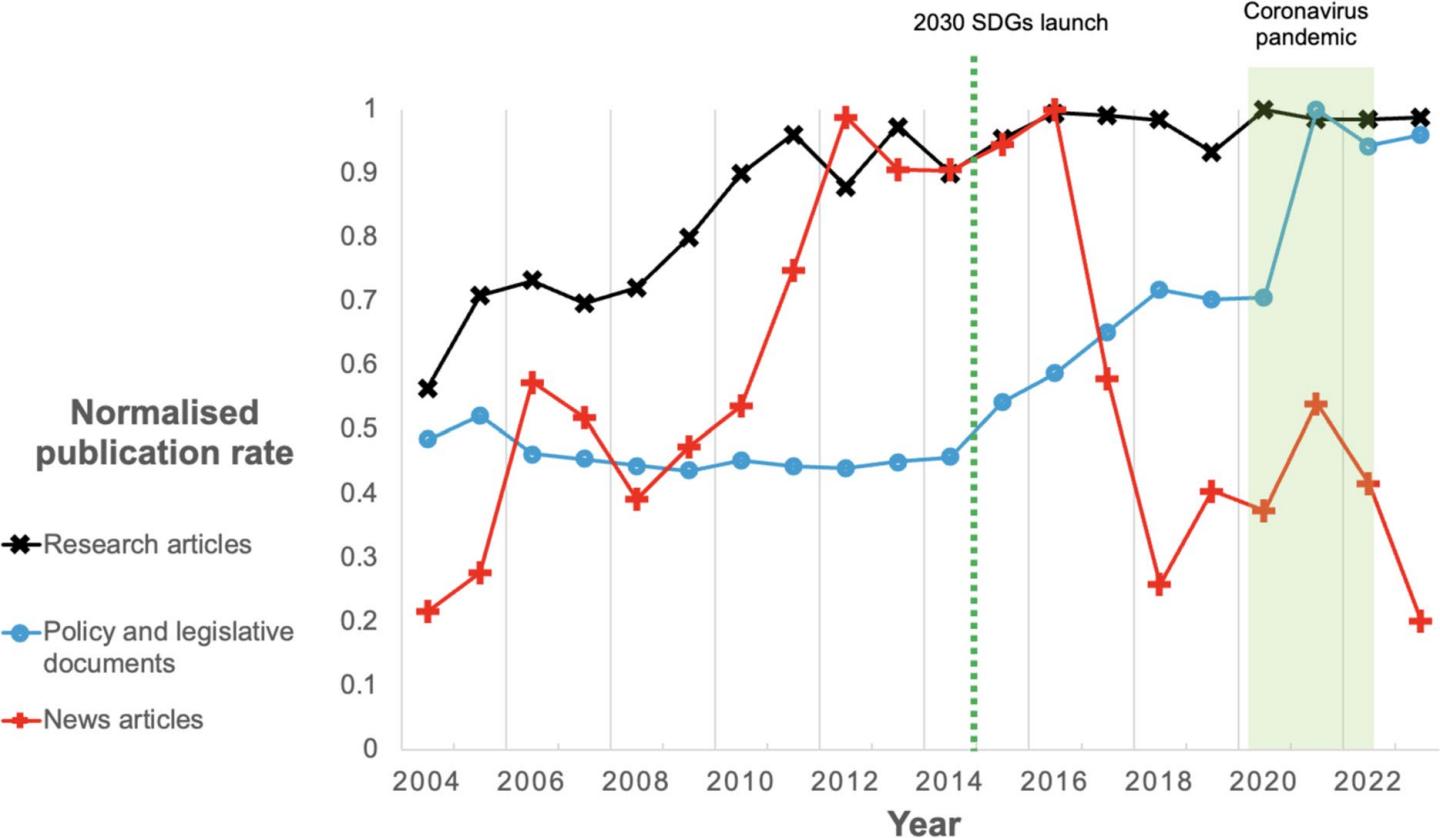
Publications on environmental hazards to and of the food system between 2004 and 2023. The graph shows the normalised publication rate for research articles, policy and legislative documents, and news articles relating to environmental hazards in the food system over the years 2004–2023. Research article data (black x) were extracted from Scopus (Elsevier, 2024), policy and legislative documents (red +) were extracted from FAOLEX (FAO, 2024), and news articles (blue o) were extracted from ProQuest (Clarivate, 2024)

There has been a broad increase in publications on environmental hazards to the food system over the period 2004–2023, but the path of this increase is not consistent across research articles, policy and legislative documents, and news articles. Academic publication rates on hazards to the food system have increased by 75% over the 20-year period, but nearly all this occurred between 2004 and 2011 (Fig. 1). In comparison, the rate at which policy and legislative documents were published was reasonably constant until around 2015, before increasing by 150% in two large jumps, the biggest around the year 2015, and one again around 2020 (Fig. 1). News articles also showed peaks in the years around 2015 and 2020 (Fig. 1), and we highlight in Fig. 1 that these periods correlate strongly with the Paris Agreement 2015 and 2030 Sustainable Development Goals launch, and the timing of the coronavirus pandemic in the years following 2020. In-depth analyses of the change in research, policy, and news over time categorised by individual hazard show the same broad trends (see Supplementary Figs. 1, 2, 3), with climate change and water scarcity in particular contributing to rises in publications, which we analyse in later sections of this paper. For all articles and documents, publication rates are normalised to 0–1 using the year. For academic articles and policy and legal documents, an additional normalisation step is performed to account for the general increase in publication rates in these fields over time (see Methods). We plotted the change in individual hazards over time, showing there was little consistent change in the relative proportions of each hazard over the 20-year period (Supplementary Figs. 1, 2, 3).

### Geographic prominence of food system hazards

**Fig. 2:**
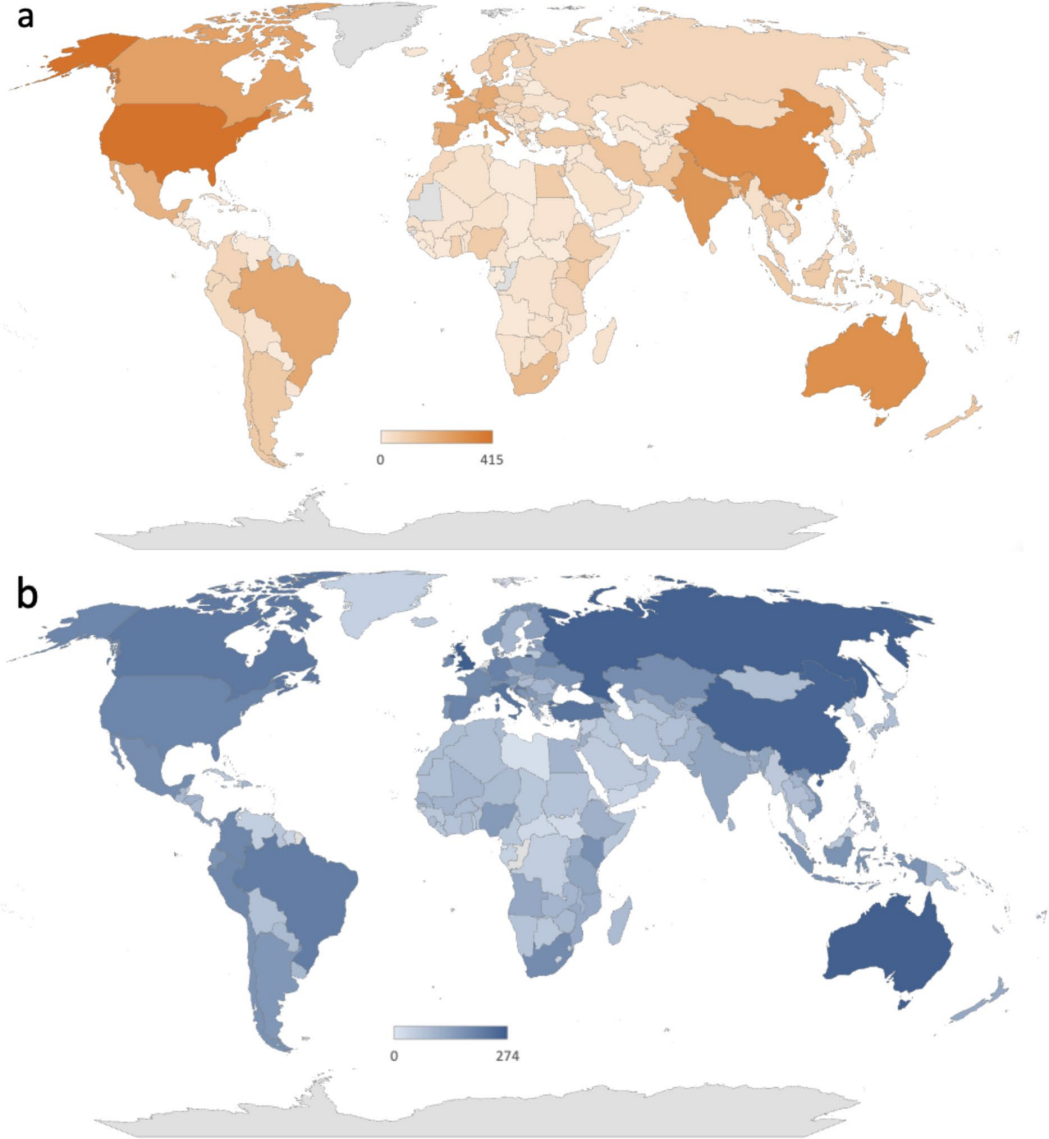
Publications on environmental hazards to the food system by country over the period 2004–2023. **a** (Orange) the number of academic articles on this topic, by country; **b** (blue) the number of policy and legislative documents on this topic, by country. Research article data were extracted from Scopus (Elsevier, 2024); policy and legislative documents were extracted from FAOLEX (FAO, 2024)

The geographical prominence of all environmental hazards in the food system by country is uneven, according to the distribution of research articles and policy and legislative documents targeted to a specific country (Fig. 2). Northern America, Western Europe, China, and Australia receive the most attention across all types of articles and documents; much less is seen in Africa. Additionally, areas with many research articles on a given hazard are not necessarily reflected in a high number of policy and legislative documents on that given hazard, and vice versa. Headline examples include India, where there is a high volume of research on environmental hazards (Fig. 2a), but relatively little policy and legislation to implement this (Fig. 2b). In comparison, Russia and Turkey both seem to produce very little hazard research, but high levels of policy; we discuss this later. For the rest of the world, research and policy and legislative documents broadly match when considering all hazards.

### Distribution of specific hazards by global sub-region

**Fig. 3:**
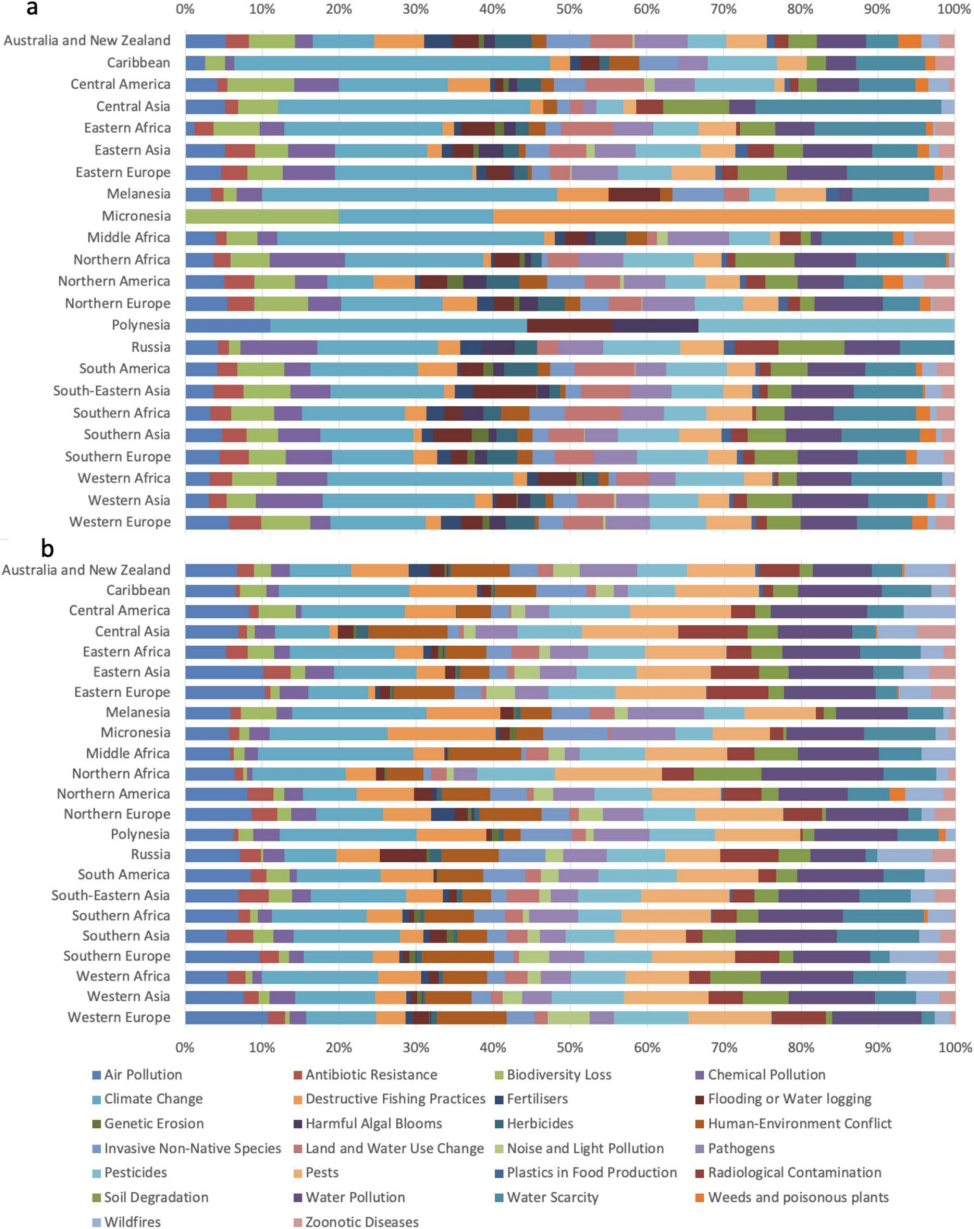
Prevalence of specific environmental hazards to the food system by global sub-region. **a** Hazard distribution according to research articles; **b** hazard distribution according to policy and legislative documents. Different colours indicate different environmental hazards to the food system, with the order of hazards in the legend following the order of presentation in the figure. Research article data were extracted from Scopus (Elsevier, 2024); policy and legislative documents were extracted from FAOLEX (FAO, 2024)

When considering specific types of hazard across global sub-regions, climate change consistently comes out as the most reported. This is shown in Fig. 3, where climate change makes up the largest single-hazard proportion of reported hazards for the majority of sub-regions, and this trend is consistent across both research and policy and legislative materials. Overall, the highest proportion of climate change–related hazards is in Middle Africa and equatorial island regions such as the Caribbean, Melanesia, and Polynesia. A key difference between the research (Fig. 3a) and policy and legislative material (Fig. 3b) is seen in smaller sub-regions such as Polynesia, Micronesia, and the Caribbean. Here, whilst policy remains fairly balanced across hazard categories, research is more unbalanced, with a very small number of publications, and focussed on a select few hazards, for example in Micronesia on biodiversity loss, climate change, and destructive fishing. In comparison, most larger sub-regions such as Eastern Asia or South America are more matched across research and policy.

### Matches and mismatches in coverage on environmental hazards

**Fig. 4:**
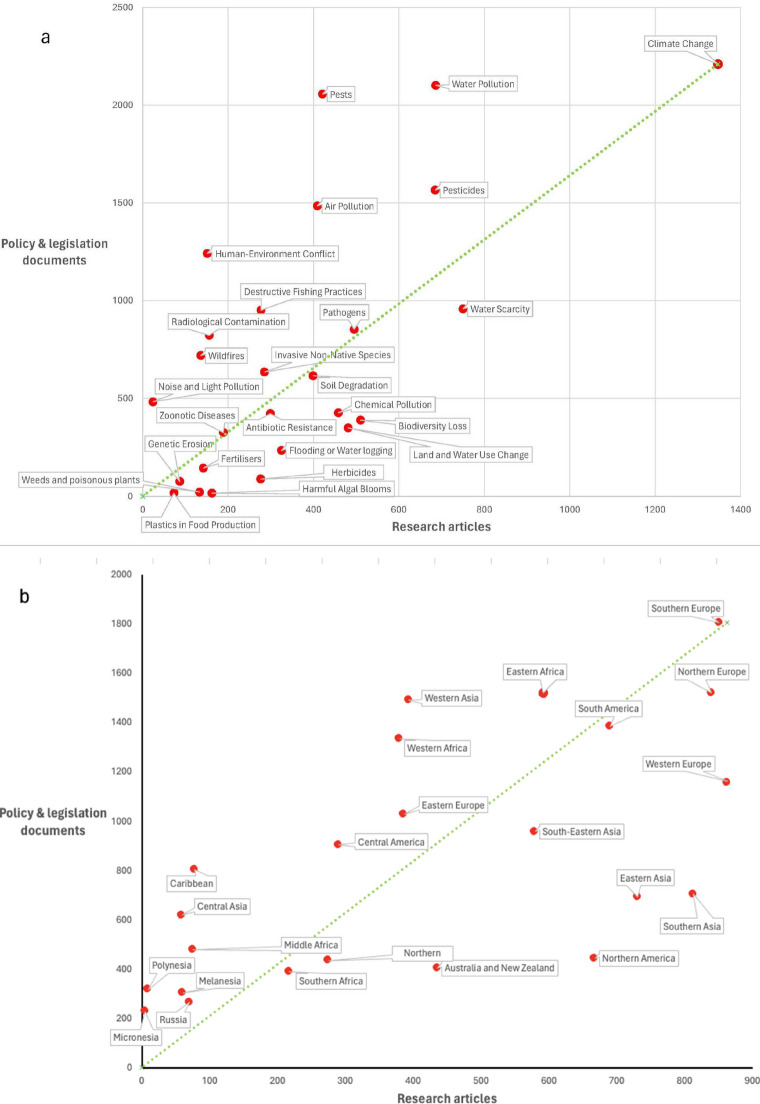

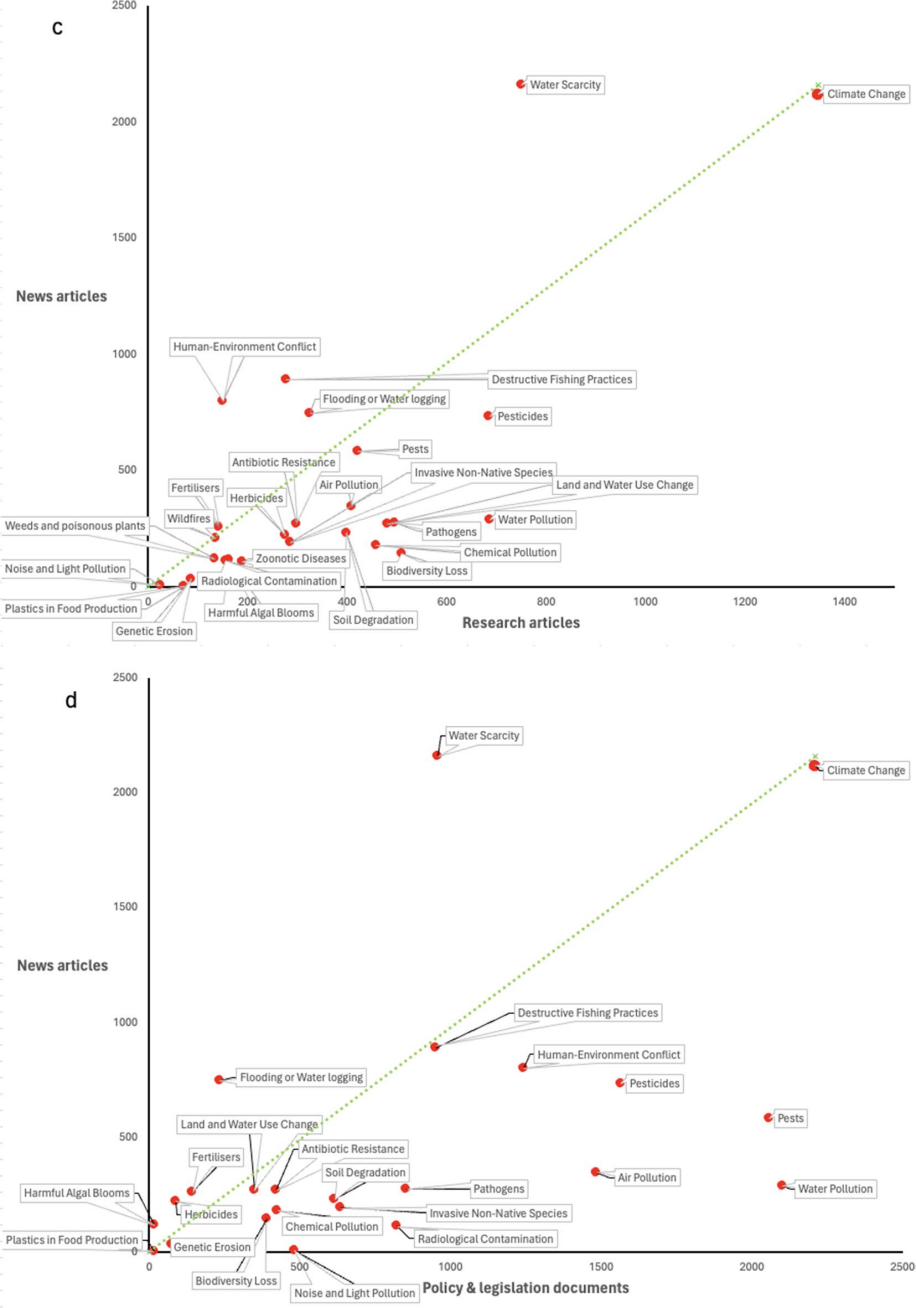
Mismatches between research articles, policy and legislation documents, and media articles across different hazards and global sub-regions. Policy and legislative coverage is compared to research coverage by hazard in **a** and by global sub-region in **b**. News coverage is compared to research coverage in **c** and to policy and legislative coverage in **d**. The numbers on the x- and y-axes refer to the number of published articles. The green dotted line represents the 1:1 matched effort line. Research article data were extracted from Scopus (Elsevier, 2024); policy and legislative documents were extracted from FAOLEX (FAO, 2024), and news articles were extracted from ProQuest (Clarivate, 2024)

When comparing reported environmental hazards to the food system across document types, clear matches and mismatches appear between research articles, policy and legislative documents, and news articles (Fig. 4). Several hazards including water pollution, pests, air pollution, and human–environment conflict receive a far greater amount of policy and legislative attention relative to the amount of research effort they receive, indicated by their position above the dotted green matched effort line (Fig. 4a). In comparison, hazards such as land and water use change and biodiversity loss receive a relatively small amount of policy coverage relative to a reasonable research effort. There are also some hazards which receive very little attention from either research or policy—including harmful algal blooms, genetic erosion, and weeds and poisonous plants—whilst other hazards like climate change receive consistently high levels of attention.

There is also a disparity by global sub-region when comparing research to policy and legislative effort (Fig. 4b). Sub-regions including Middle Africa, East Africa, Western Africa, and Polynesia are all relatively light on research but policy rich across all hazards, whereas regions including Northern America, Southeast Asia, and Western and Northern Europe are all heavier on research but lighter on policy—we reflect later on how there may be income or development related reasons underpinning this difference.

Comparing news coverage with that covered by research and policy draws out further nuances in coverage (Fig. 4c, d). Climate change, water scarcity, and flooding consistently receive the most attention from the media across hazards relative to the amount of research or policy and legislative effort they receive. Other hazards such as invasive non-native species and soil degradation receive much less attention from the media relative to the amount of research and policy effort received.

## Discussion

This study highlights and discusses several critical themes in the way in which food system hazards are presented globally in the different literature sources. Academic, policy and legislative, and media attention on environmental hazards to the food system have been increasing over time, but notable increases in attention correspond strongly with major global geopolitical events. Geographical coverage on hazards by research and policy is not evenly distributed, and even within sub-regions, the balance of research and policy does not always match. A small number of hazards—notably climate change, water scarcity, and water pollution—receive a dominating level of attention, whereas other hazards receive very little focus, particularly from the media, despite representing very important hazards in terms of their impacts, for example harmful algal blooms which cause an estimate US $8 billion in annual losses to aquaculture industry, equivalent to 3.2% of the global annual revenue of aquaculture (Lenzen et al., 2021).

We observed that attention on environmental hazards by research, policy and legislation, and news increased over the period 2004–2023, and that for policy and legislation and news, this correlated with major geopolitical events, notably the launch of the SDGs in 2015 and the coronavirus pandemic. The increase in media and policy attention on environmental hazards such as destructive fishing in correlation with the 2015 Paris Agreement has been reported in other studies (Willer et al., 2022), and the role of coronavirus in increasing both public and political awareness and action on the issues posed by environmental change is also well documented (Chakraborty & Maity, 2020). There may also be other global events or factors that contributed to these two peaks in attention. Particularly, when considering policy, this falls in line with other studies that highlight the power of international events in driving responses to climatic change (Hase et al., 2021), and it signals that policy and legislation can be influential in setting the media (and, by inference, broader public) agenda on environmental causes. There is however still a need for more and improved data, and as authors, we emphasise the importance or further work in this field on the strength of the correlation between the presence of hazards in research, policy and media, and the real-world prevalence of hazards.

Geographic coverage by research and policy or legislation on environmental hazards is not evenly distributed on a global level. Northern America, Western Europe, China, and Australia receive the most attention, maybe reflective of the greater economic and infrastructure resources for both research and policy implementation (Allik et al., 2020; Jaffe et al., 2020). However, there is also a great deal of nuance in the distribution. Areas such as the Indian subcontinent have a high level of research performed in relation to environmental hazards, but relatively little in the way of policy implementation—perhaps reflective of the fast rate of development in this region and a more relaxed legislative system and still growing climate policy sector (Mohan & Wehnert, 2019). Russia shows the opposite trend—high levels of policy but relatively little as regards to publicly available research. This may be reflective of a variety of factors, including funding access and the long shadow of the ‘brain drain’ of the 1990s, alongside potential high levels of data accessibility restrictions (Clery, 2010). The more in-depth comparison of research and policy focus in Fig. 4b highlights how there may be a broad-scale development or income-related divide in research and policy effort. Higher-income regions such as Northern America, Southeast Asia, and Western and Northern Europe are all relatively research heavy but comparatively policy light, whilst regions still with high levels of development potential such as Middle Africa, East Africa, West Africa, and Polynesia are heavier on policy but lighter on research. This may be reflective of the influence of global policies at a national level whilst research is slower to follow. This issue has been highlighted in recent studies, which call for a need to increase funding for Africa-based research in order to strengthen responses to environmental change (Overland et al., 2022).

When examining specific environmental hazards across sub-regions, the clear trend that emerges is a dominating focus on climate change and water issues, with much less focus on other issues including biodiversity loss and genetic erosion, highlighted in both Figs. 3 and 4. A heavy focus on climate change is justified, and it is the driving force behind many other hazards, but this should not be at the expense of tackling other hazards (Ortiz et al., 2021). This growing global focus on climate issues potentially at the expense of biodiversity was flagged by researchers in 2014 (Veríssimo et al., 2014), and our study suggests that this trend has continued and there is a need to try and drive change here, particularly given that climate and biodiversity are so tightly linked. We highlight how for smaller population sub-regions such as Micronesia and Polynesia, research efforts are often more unbalanced and focussed on a narrower range of hazards, perhaps reflective of careful prioritisation of resources, people time, and space, whilst the global policies in force in these areas appear to remain balanced.

Critical areas of nuance emerge when contrasting the relative emphasis of research, policy, and the news on specific hazards. The media gives relatively minimal focus on soil degradation, pesticides, and invasive non-native species relative to the amount of political and legislative effort they receive. This may reflect how these types of hazards are often difficult to build into newsworthy headlines, despite having important and often growing impacts on food production (Bouma, 2019; Herrick et al., 2013). A contrast to this is flooding and waterlogging, which is given a high level of coverage by the media, perhaps reflective of the impact of such events directly on people, the deadly rains and floods of April 2022 in South Africa being one example (Bouchard et al., 2023). When comparing research and policy, a headline finding is how hazards including human–environment conflict (i.e. interactions between humans and wild fauna or flora with native outcomes (König et al., 2020)), destructive fishing, and pests receive relatively more policy attention than the amount of research effort. This may reflect the more visibly immediate economic and social impacts of these forms of hazard— for example on West African fisheries where decline of commercial fish stocks can drive millions into devastating poverty with a domino effect on the economy (Asiedu et al., 2021), relative to other hazards such as land use change, biodiversity loss, and invasives which may be ‘slower burners’ regarding their impact and effectively become ‘invisible’ despite the threat of anthropogenic mass extinction (Crist, 2022).

## Conclusion

Overall, there is a wealth of valuable data available in research, policy, and legislative and news databases, which can help inform current and future conservation decision making actions. We have explored how major geopolitical events can be important in driving focus on environmental hazards, and how for hazards such as climate change and water scarcity the effects are clearly visible, with large and increasing amounts of attention over the past two decades. However, we emphasise the importance of not letting the less conspicuous hazards—such as biodiversity loss or novel invasive species—slip by with minimal attention. The consequences of neglect in our policy and research activities could be severe and irreversible. Even in small island nations like the Galapagos, novel invasives have been difficult to control, impossible or incredibly costly to eradicate, and historically have caused numerous extinctions (Ballesteros-Mejia et al., 2021)—a parallel challenge on a global level would be overwhelming. There is a call for careful consideration of all hazards affecting the global food system in order to inform effective management strategies for the future.

## Supplementary Information

Below is the link to the electronic supplementary material.

ESM 1 (PDF 144 KB)

## Data Availability

All data are available in the manuscript or supplementary materials.

## Declarations

All authors have read, understood, and have complied as applicable with the statement on ‘Ethical responsibilities of Authors’ as found in the Instructions for Authors.
