# Supplementary material for: Mapping hazards to the global food system
Source: Environ Monit Assess. 2024 Dec 4;197(1):18. doi: 10.1007/s10661-024-13475-4 (PMC11618188; doi:10.1007/s10661-024-13475-4)
Supplement: Supplementary file 1 — (PDF 144 KB) [file 10661_2024_13475_MOESM1_ESM.pdf]

Supplementary Data

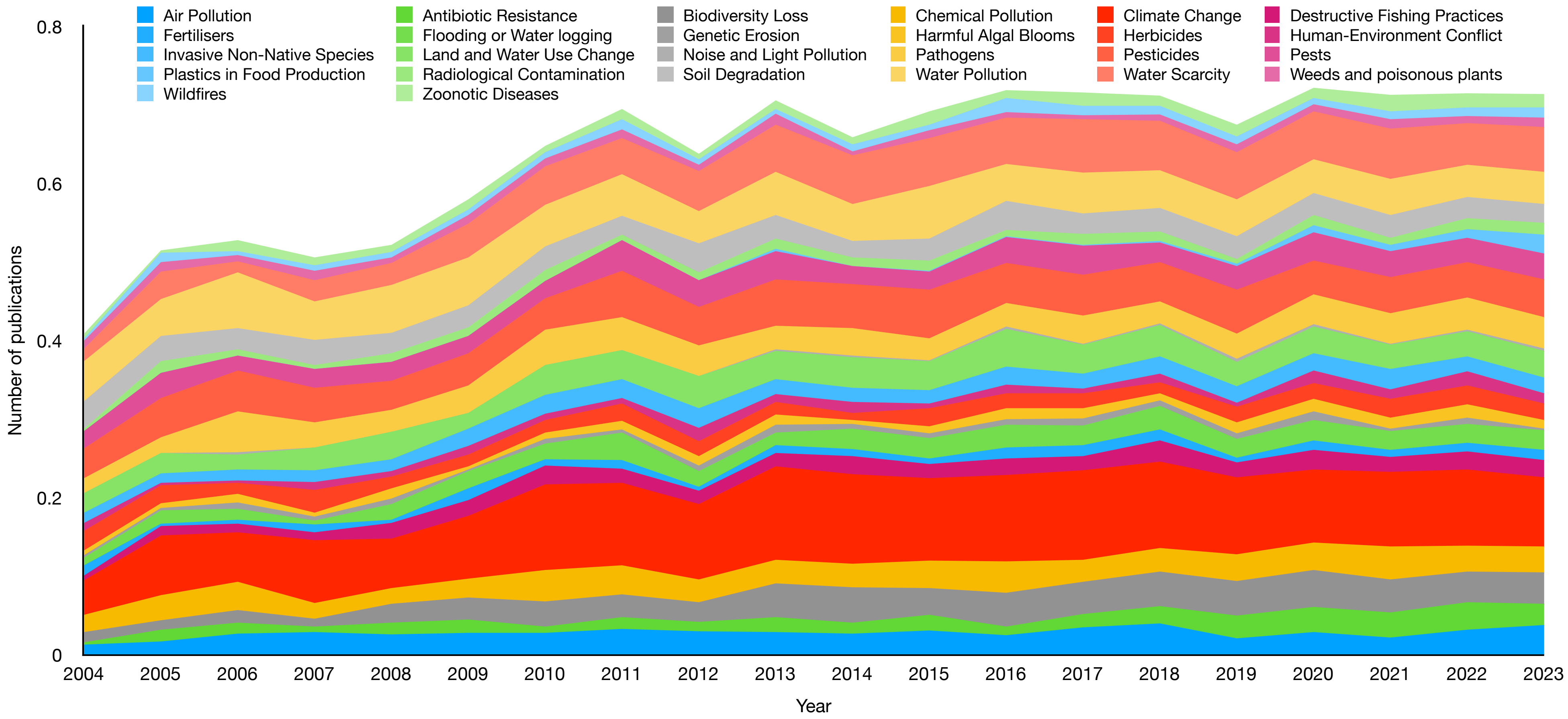

Supplementary Figure 1. Research publications on environmental hazards to and of the food system between 2004-2023, broken down by hazard type. Different colors represent different hazards. Research article data were extracted from Scopus.

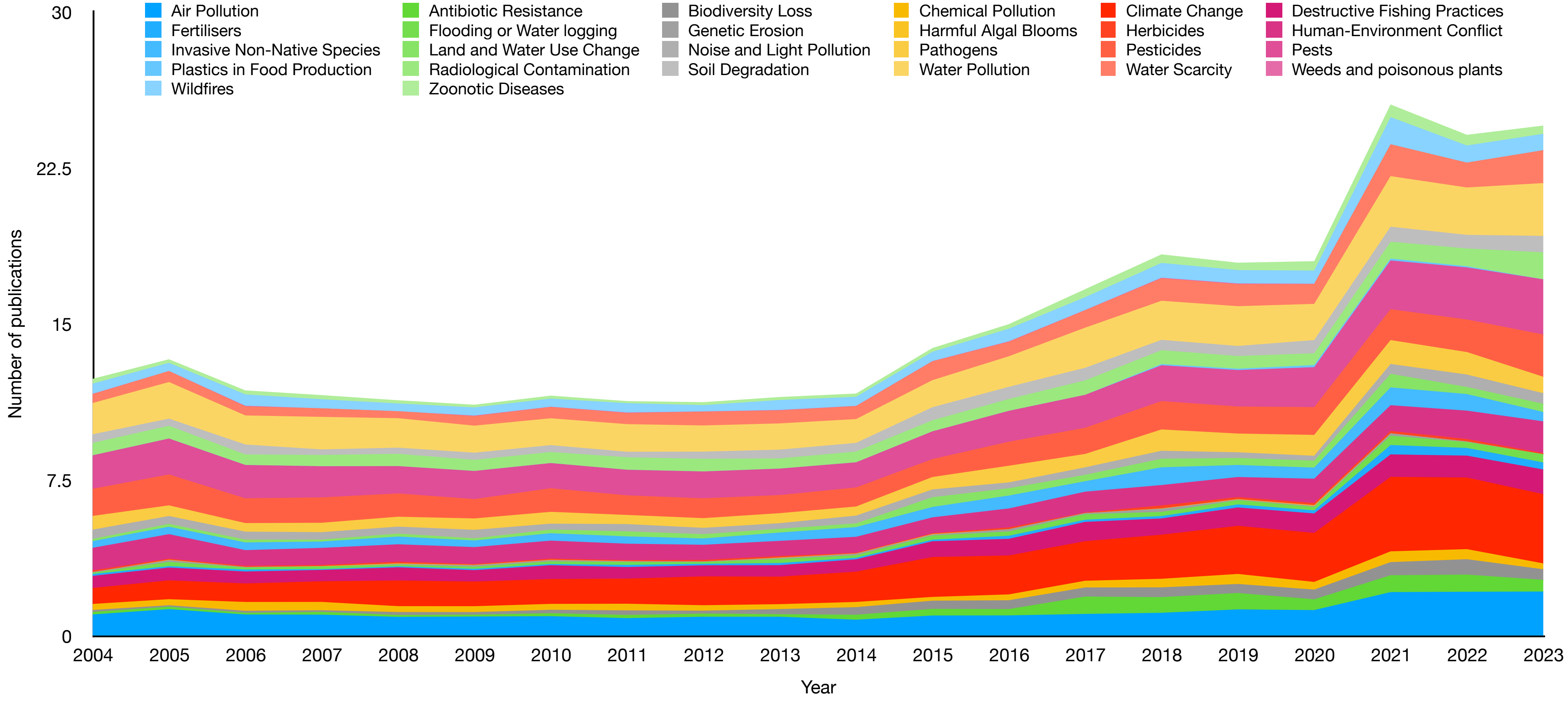

Supplementary Figure 2. Policy and legislative publications on environmental hazards to and of the food system between 2004-2023, broken down by hazard type. Different colors represent different hazards. Policy and legislative data were extracted from FAOLEX.

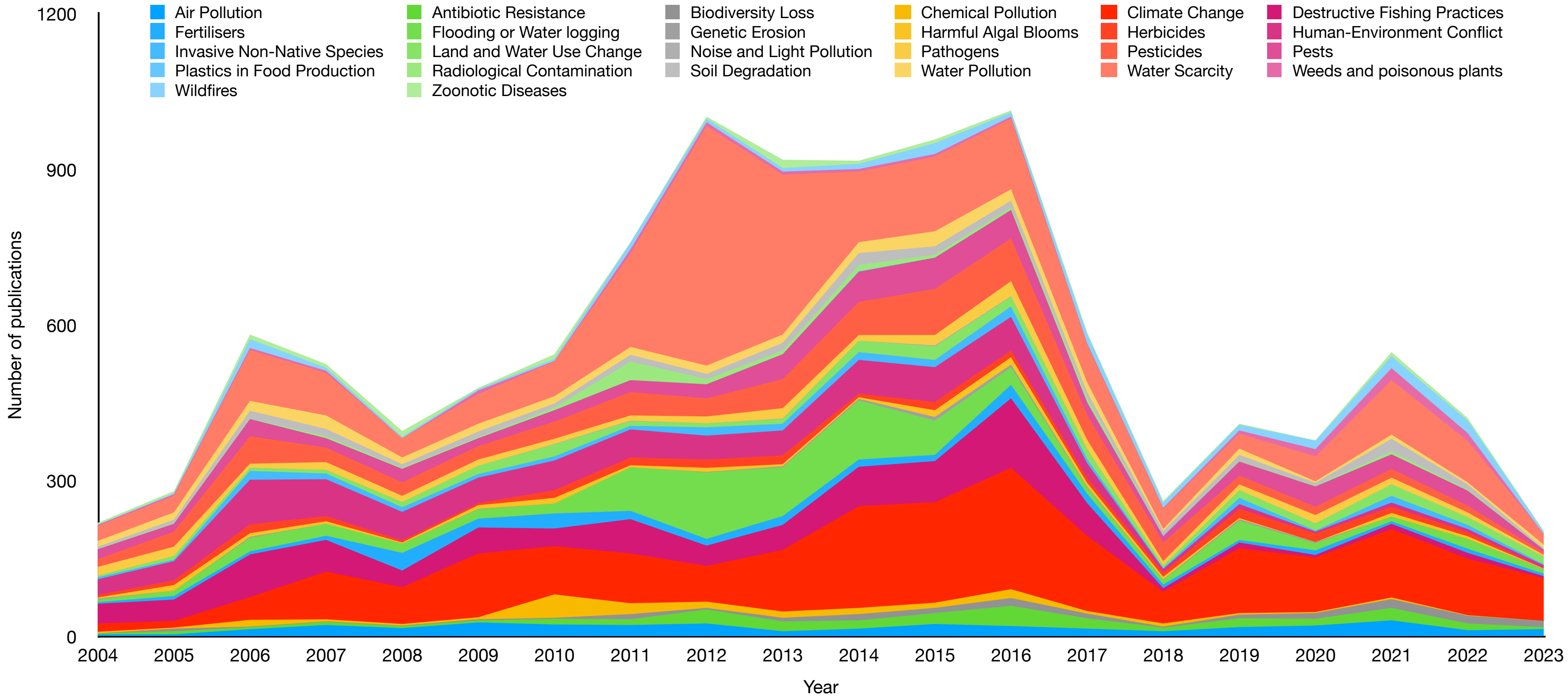

Supplementary Figure 3. Media publications on environmental hazards to and of the food system between 2004-2023, broken down by hazard type. Different colors represent different hazards. Media data were extracted from ProQuest.

Supplementary Table 1. Hazard sub-search terms. Table lists the sub-search terms used for each of the 26 hazards.

#	Category	HAZARD	Sub-Search Term 1	Sub-Search Term 2	Sub-Search Term 3	Sub-Search Term 4	Sub-Search Term 5	Sub-Search Term 6
1	Biological	Antibiotic Resistance	Antibiotic resistance	Livestock feed additives	Resistant bacteria	Drug residues	One Health	Antimicrobial
2	Biological	Biodiversity Loss	Biodiversity loss	Species extinction	Wildlife decline	Ecosystem services	Conservation biology	Range shift
3	Biological	Genetic Erosion	Genetic erosion	Monoculture risk	Crop diversity	Heirloom varieties	Seed banks	Agrobiodiversity
4	Biological	Harmful Algal Blooms	Algal blooms	Nutrient loading	Blue-green algae	Toxic algae	Cyanobacteria	Red tide
5	Biological	Human-Environment Conflict	Human-environment conflict	Human-wildlife conflict	Hunting	Poaching	Organism extraction	Land use conflict
6	Biological	Invasive Non-Native Species	Invasive species	Non-native species	Alien species	Biocontrol	Invasive plant	Biofouling
7	Biological	Pathogens	Pathogen	Plant disease	Fungal pathogen	Disease vectors	Pathogen surveillance	Biosecurity
8	Biological	Pests	Pests	Integrated pest management	Biological pest control	Crop pests	Rodents	Insects
9	Biological	Weeds and poisonous plants	Weeds and poisonous plants	Weed control	Invasive weeds	Allelopathy	Weed ecology	Poisonous plants
10	Biological	Zoonotic Diseases	Zoonotic disease	Animal to human transmission	Infectious diseases	Outbreak surveillance	Wildlife reservoirs	
11	Chemical	Air Pollution	Air pollution	Ozone depletion	Particulate matter	Greenhouse gases	Methane emissions	Refrigerant
12	Chemical	Chemical Pollution	Chemical pollution	Industrial chemicals	Persistent organic pollutants	Oil spill	Heavy metals	Detergents
13	Chemical	Fertilisers	Fertiliser	Nitrate pollution	Phosphorus runoff	Fertiliser leaching	Soil acidification	NPKS
14	Chemical	Herbicides	Herbicides	Glyphosate	Atrazine	Herbicide resistance	Non-target species	
15	Chemical	Pesticides	Pesticides	Organochlorides	Neonicotinoids	Pesticide resistance	Bioaccumulation	Pollinator decline
16	Chemical	Plastics in Food Production	Plastics in food production	Plastic residues	Soil microplastics	Plastic pollution	Microplastic accumulation	Microplastics in seafood
17	Chemical	Radiological Contamination	Radiological contamination	Nuclear fallout	Radiation	Food irradiation	Radioactive contamination	
18	Chemical	Water Pollution	Water pollution	Agricultural runoff	Industrial effluents	Sewage	Wastewater discharge	Eutrophication
19	Physical	Climate Change	Climate change	Extreme temperature	Extreme weather	Sea level rise	Ocean acidification	Global warming

20	Physical	Destructive Fishing Practices	Destructive fishing practices	Bycatch	Overfishing	Bottom trawling	Marine protected areas	Illegal fishing
21	Physical	Flooding or Water logging	Flooding or water logging	Floodplain management	Flooding	Water logging	Sediment deposition	Salinity incursion
22	Physical	Land and Water Use Change	Land use change	Urbanisation	Deforestation	Wetland drainage	Habitat fragmentation	Irrigation practices
23	Physical	Noise and Light Pollution	Noise pollution	Light pollution	Sound pollution	Dark skies	Acoustic ecology	
24	Physical	Soil Degradation	Soil degradation	Topsoil loss	Desertification	Salinization	Soil compaction	Soil erosion
25	Physical	Water Scarcity	Water scarcity	Aquifer depletion	Overdrafting	Water footprint	Drought	Water security
26	Physical	Wildfires	Wildfires	Forest fire	Prescribed burns	Fire management	Post-fire restoration	Bushfire
